# Oligonucleotide-Templated Reactions for Sensing Nucleic Acids

**DOI:** 10.3390/molecules17032446

**Published:** 2012-02-29

**Authors:** Aya Shibata, Hiroshi Abe, Yoshihiro Ito

**Affiliations:** Nano Medical Engineering Laboratory, RIKEN Advanced Science Institute 2-1, Hirosawa, Wako-Shi, Saitama 351-0198, Japan; Email: ashibata@riken.jp (A.S.); y-ito@riken.jp (Y.I.)

**Keywords:** detection, amplification, fluorescence, DNA, protein nucleic acid, RNA

## Abstract

Oligonucleotide-templated reactions are useful for applying nucleic acid sensing. Various chemistries for oligonucleotide-templated reaction have been reported so far. Major scientific interests are focused on the development of signal amplification systems and signal generation systems. We introduce the recent advances of oligonucleotide-templated reaction in consideration of the above two points.

## 1. Introduction

Oligonucleotide-templated reactions (OTRs) are powerful tools for the detection of nucleic acid sequences [1,2]. Basically, two probes that carry a reactive group bind to the template strand located in next each other and a chemical reaction proceeds on the template without any enzyme or additive reagent. This reaction can be controlled and accelerated by effective concentration, as the reaction proceeds only in the presence of the template. As a result, the existence of target oligonucleotide sequences can be detected by the presence of a signal resulting from the chemical reaction. Various chemistries for OTRs have been reported to date. The design of signal amplification mechanisms and fluorogenic systems as diagnostic methods is exciting scientifically and also important practically. In this review, we present the recent advances in the development of OTR probes that are applied to oligonucleotide detection.

## 2. The Templated Photochemical Reaction

The DNA-templated photochemical reaction was reported for the first time in 1982 by Lewis and Hanawalt [3]. The DNA-templated photoligation was achieved by induced cyclobutene dimer formation between the termini of dT_10_ oligonucleotides in the presence of a poly(A) template >290 nm UV light is preferable to perform effective ligation. However, this condition entailed a risk of dimerization at other sites [4]. Therefore, templated photoligation methods using coumarin [5], psoralen [6], and stilbene [7,8] as the photoactivation reagent were developed. Furthermore, Liu and Taylor reported a photoligation method using 4-thiothymidine activated at 366 nm, which is a wavelength that does not induce damage to DNA or RNA [9,10,11]. Photoligation products were obtained in the presence of template by using this probes, with a yield of 40% [11].

**Figure 1:**
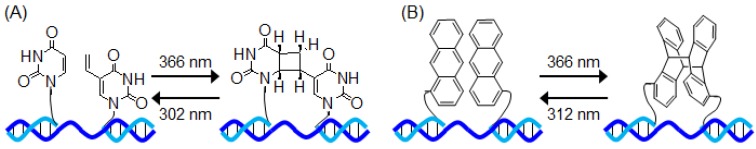
Reversible photoligation. (**A**) [2+2] cycloaddition and (**B**) [4+4] cycloaddition.

Fujimoto and coworkers reported a new system that used 5'-vinyldeoxyuridine as a photoactivation reagent (Figure 1A) [12,13,14]. This molecule is also activated by irradiation at 366 nm. In the presence of template, the photoactivated 5'-vinyldeoxyuridine established a link with a pyrimidine of the terminal of an adjacent probe by [2+2] cycloaddition. Intriguingly, this [2+2] cycloaddition linkage was cleaved by 1 h irradiation at 302 nm and the ligation product reverted to 5'-vinyldeoxyuridine and pyrimidine. Therefore, these authors succeeded in developing a reversible photoligation method by switching between irradiation at two wavelengths. Furthermore, they improved the photoactivation reagent and reported a photoligation reaction using 5'-carboxyvinyldeoxyuridine [15,16,17,18,19,20,21]. The irradiation of these probes at 366 nm for 1 h in the presence of template resulted in the expected ligation product, with a yield of 93%. These authors applied these probes to the detection of single-base mismatches [19,22,23,24,25], the formation of various special structures [13,14,15,17,21,26,27], the site-specific transition of cytosine to uracil [16,28,29], and the development of DNA computing [18].

Ihara and coworkers reported [4+4] cycloaddition on the template DNA using anthracene-DNA conjugate probes (Figure 1B) [30,31,32,33]. Anthracene also forms a photodimer after irradiation at 366 nm [34,35], Although photoligation using these anthracene probes was completed after 1 min of irradiation in the presence of template, the ligation products were not obtained in the presence of single-base mismatches. Moreover, anthracene-functionalized probes were applied to the construction of high-order DNA structures [33,36].

**Figure 2:**
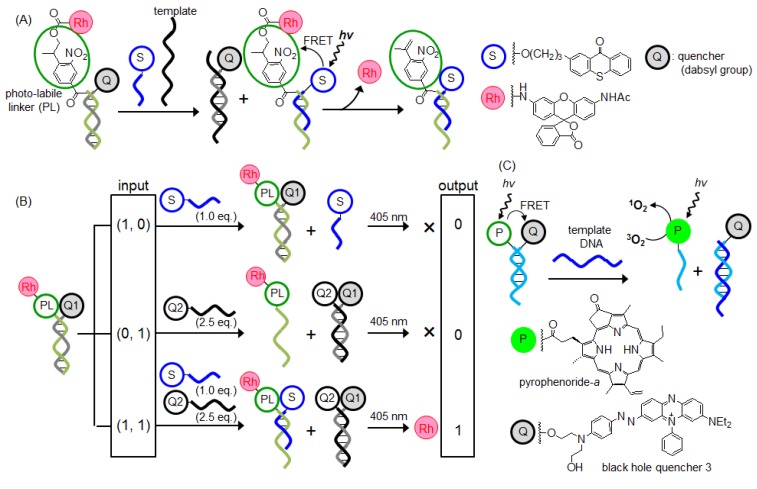
Schematic representation of (**A**) the photorelease system based on the energy transfer from the sensitizer to the linker; (**B**) the logic gate (AND gate); and (**C**) the ^1^O_2_ generation system based on a templated photoactive reaction.

Photoactive groups are used as protection groups of the caged compounds of various bioactive molecules, or as reporters [37]. Several groups reported the molecular releasing system by utilizing photo chemical reaction [38,39,40,41,42,43,44]. These systems are based on energy transfer between a quencher or sensitizer and the photoactive group. Tanabe and coworkers reported a photoactive drug-release system based on the molecular beacon (MB) strategy [38,39]. Although the photoreaction was very inefficient when the MB was in the closed form structure and in the absence of template, irradiation at 312 nm of the open form structure hybridized with the target DNA resulted in the rapid release of the drug from the MB. Winssinger and coworkers reported a photo-releasing system that is activated by irradiation at 405 nm (Figure 2A) [40]. They successfully constructed logic gates using four components: quencher 1, 2, photoactivator, and sensitizer (Figure 2B). In addition, Gothelf and coworkers reported for the first time the on/off switching of a ^1^O_2_ generation system based on a templated photoactive reaction (Figure 2C) [41,42,44]. This system is expected to be applied to photodynamic therapy.

## 3. The Templated Ester-Hydrolysis Reaction

**Figure 3:**
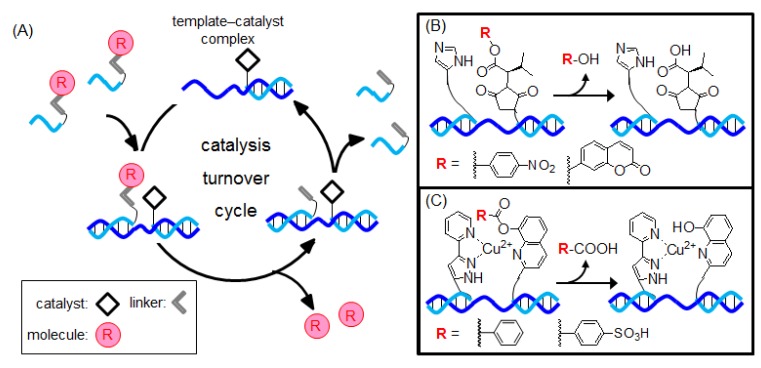
(**A**) Molecular release via templated catalysis; (**B**) organocatalyzed ester hydrolysis reaction; and (**C**) metal-catalyzed hydrolysis reaction.

In 2000, Taylor and coworkers reported the templated ester-hydrolysis reaction by utilizing imidazole as a catalyst (Figure 3) [45]. This method was the first example of a drug-releasing system based on genetic information. This system consists of two probes: one probe has an imidazole group at the 5' terminal, whereas the other probe is esterified to *p*-nitrophenol or coumarin at the 3' terminal. In the presence of the template, the imidazole group of the probe hydrolyzes the phenyl ester bond of the other probe (Figure 3B). After the reaction, the complex formed between the catalytic probe and the template is reused in a new reaction. This complex behaved like enzymes and carried out multiple turnover of molecular release. Taylor and coworkers examined this system using DNA [45,46] or peptide-nucleic acid (PNA) [47,48] probes and reported that each probe exhibited high selectivity (*i.e.*, single-base mismatch).

Metal catalysis is generally more efficient than organocatalysis. Therefore, Kraemer and coworkers reported a new releasing system using templated metal catalysis (Figure 3C) [49,50,51]. Their system consists of two PNA probes: one probe has an ester substrate at the N terminal, whereas the other probe has a copper(II) chelating pyridylpyrazolyl group at the C terminal. When two PNA probes were brought into close proximity at the template, the carboxylate substrate was released via a metal-catalyzed ester hydrolysis reaction. A turnover number of 35 was achieved with a full-match template [49]. Conversely, the reactivity of the hydrolysis reaction decreased by ~100 times in the presence of a single-base mismatch template [51].

## 4. The Templated Nucleophilic Substitution Reaction

**Figure 4:**
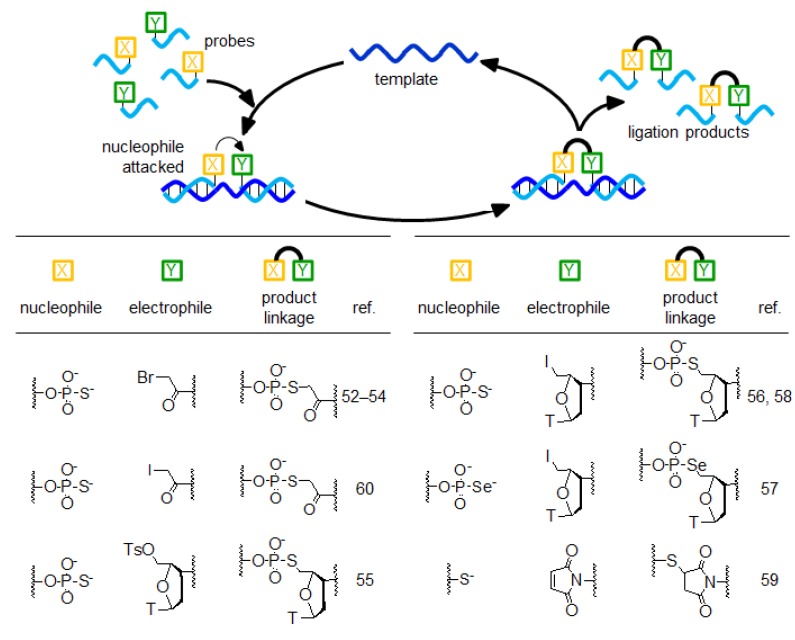
Nucleophilic substitution reaction-mediated autoligation.

Some research groups have reported templated chemical ligation based on a nucleophilic substitution reaction (Figure 4) [52,53,54,55,56,57,58,59,60]. These systems consist of two DNA probes that bind to target oligonucleotides located in close proximity: One probe has an electrophilic group at the 5' terminal, whereas the other probe has a nucleophilic group at the 3' terminal. In the presence of templates, the nucleophilic group attacks the electrophilic group and forms a new linkage. Phosphorothioate, phosphoroselenoate, or thiol is used as the nucleophilic group, and haloacetyl, maleimide, tosyl, or 5'-iodothymidine is used as the electrophilic group. In particular, in the case of the combination of the phosphorothioate and iodoacetyl groups, the reaction speed is faster than that of other combinations and the reaction is almost completed within 1 min [60]. On the other hand, the linkage resulting from the ligation reaction between phosphorothioate and tosyl [55] or 5'-iodothymidine [56,57,58] groups resembles the natural phosphodiester linkage in form. These probes exhibit high selectivity and are used in the detection of single-base mismatches in templates.

**Figure 5:**
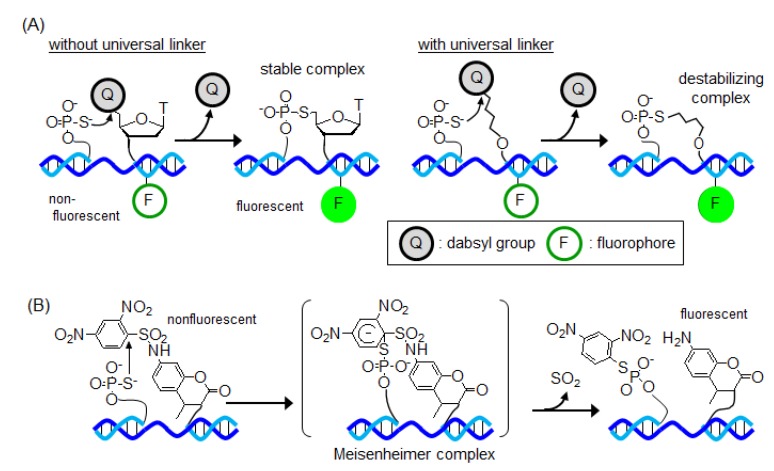
Fluorescence signal-generating method based on (**A**) S_N_2 reaction or (**B**) S_N_Ar reaction.

Kool and coworkers reported the quenched autoligation (QUAL) probe based on an S_N_2 reaction as a fluorescence-signal-generating method (Figure 5A) [61,62,63,64]. The electrophilic probe has dabsyl as a quencher and leaving group at the 5' terminal. In the absence of the template, the fluorophore and quencher molecules are positioned close to each other and fluorescence resonance energy transfer (FRET) occurs. The hybridization of the QUAL probes to the target template leads to the attack of the 5' carbon of the electrophilic probe by the phosphorothioate group of the nucleophilic probe, which results in ligation of the probe and unquenching of the fluorophore. The reaction of QUAL probes in the presence of target DNA exhibited good discrimination ability in single nucleotide level, dropping in reactivity by 35-fold compared with the full-match template [61]. Furthermore, this probe successfully detected 16S rRNA in living bacterial cells [62]. The QUAL probe can be used easily to perform multicolorization by changing the fluorophore. As a result of the detection of single-base mismatch in each template using four different QUAL probes, four fluorescence signals corresponding to the probe and template were obtained [63]. The products of the templated ligation reaction strongly inhibited the next catalysis reaction. Therefore, the authors developed new QUAL probes containing a universal linker at the 5' terminal [65,66,67,68,69]. This strategy aimed at the destabilization of the ligation product by introducing a flexible linker into the linkage. The comparison of the stability of duplexes containing the universal linker with that of duplexes without the linker revealed that *T*_m_ was decreased by about 12 °C. Product inhibition was improved by the destabilization of the ligation product and the turnover number reached up to 92 [65]. Furthermore, this QUAL probe succeeded in the detection of mRNA in living bacterial [67,68,69] and human [66] cells.

Abe, Ito and coworkers reported a nonligation-type fluorogenic sensing system based on a nucleophilic aromatic substitution (S_N_Ar) reaction (Figure 5B) [70]. This system consists of two DNA probes: One probe has an electrophilic 2,4-dinitrobenzenesulfonyl-protected 7-amino-4-methyl-3-coumarinylacetic acid dye at the 3' terminal, whereas the other probe has a nucleophilic phosphorothioate group at the 5' terminal. The phosphorothioate group attacked by the dinitrobenzene group of the coumarin derivative forms a Meisenheimer complex, as an intermediate, on the template. This complex quickly decomposes to give an unmasked amino group on the coumarin, accompanied by the transfer of a dinitrobenzene group to the phosphorothioate probe. Thereby, the probe emits a fluorescence signal without ligation between the probes.

## 5. The Templated Staudinger Reaction

**Figure 6:**
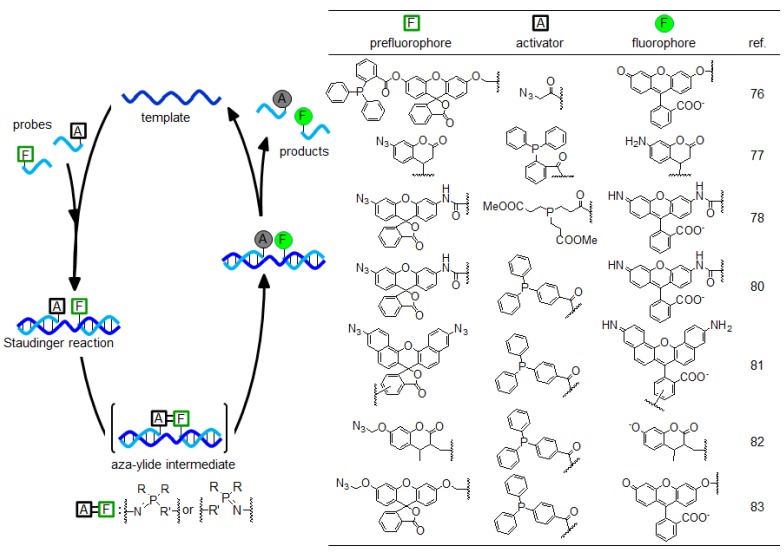
Fluorescence signal-generating method triggered by the Staudinger reaction.

The azido group has been used in various biochemical reactions, such as the Staudinger reaction or click chemistry [71,72,73,74,75]. The fluorescence turn-on system, which is triggered by templated reduction of azido groups, was reported for the first time by Taylor and coworkers (Figure 6) [76]. This system consists of two PNA probes: One probe has a triphenylphosphine (TPP) masked fluorescein at the N terminal, whereas the other probe has an azido group at the C terminal. In the presence of the template, the Staudinger reaction proceeds between the TPP group of one probe and the azido group of the other probe, leading to cleavage of the ester bond of fluorescein and the generation of fluorescence signals.

Subsequently, reduction-triggered fluorescence probes were reported by Winssinger and coworkers [77,78,79] and Abe and coworkers [80,81]. Their system involved the reaction between the azido group of a fluorophore and the reducing reagent (TPP [77,80,81] or tris(2-carboxyethyl) phosphine (TCEP) [78,79] ) on the template, with generation of fluorescence after reduction of the azido group (Figure 6). These probes exhibited a high signal-to-background (S/B) ratio and successfully detected mRNA in living bacterial [80] or human [78] cells. Furthermore, Winssinger and coworkers used these probes to detect and quantify micro RNA in fixed human cells [79].

The reaction between the azido probe and the TPP probe initially produced the aza-ylide intermediate and then the intermediate is hydrolyzed. Because this aza-ylide bond was stable, the possibility that this intermediate prevents catalytic turnover was indicated [80]. To obtain a high catalytic turnover in the templated reaction, the azidomethyl group was used as the new protection group (Figure 6) [82,83,84,85]. The reaction between the azidomethyl probe and TPP in the presence of the template was completed in 3 min and the turnover number obtained was 22 (in 30 min) [82] or 54 (in 4 h) [83]. In addition, Abe, Ito and coworkers detected a lariat RNA structure *in vitro* [85] and quantified mRNA in living human cells [83].

These azido-masked fluorogenic molecules offered very low background signal. For example, the S/B ratio of azidomethyl-masked fluorescein is 300 fold [83]. In contrast, in the case of the FRET mechanism, the quenching efficiency reaches a maximum of only 98% and the corresponding S/B ratio is only 50 fold [86]. Thus, the S/B ratio of these fluorogenic molecules could exceed that of the FRET mechanism.

One problem of the Staudinger reaction is the oxidation of the phosphine group under biological conditions. Therefore, usually, the templated Staudinger reaction is performed in the presence of excess phosphine probe *vs*. azido probe. Winssinger and coworkers reported the use of a metal-catalyzed photoreduction probe as a method to improve this problem [87]. Their reduction probe had a [Ru(bpy)_3_]^2+^ analog instead of a phosphine group. The azido-reduction reaction using the Ru^2+^ catalyst is induced by irradiation using visible light [88]. They reported that the reaction was carried out efficiently with a 2% volume of the Ru^2+^ probe.

The templated Staudinger reaction is used not only as the fluorescence off/on control, but also as a trigger of the release of the molecule based on genetic information. Kool and coworkers reported a quenched Staudinger-triggered α-azidoether release (Q-STAR) probe for use in the releasing system [89,90,91,92]. The α-azidoether linker connects the quencher with the DNA probe, and the quencher is released when the α-azidoether linker is cleaved as a result of the Staudinger reaction on the template. The Q-STAR probe exhibited high selectivity (a single-base mismatch in the target decreased its reactivity by 120 times) and signal amplification (turnover > 75) [89]. In addition, the Q-STAR probe recognized a single-base difference in 16S rRNA and discriminated between *E. coli* and *S. enterica* [89,92]. Moreover, the authors applied the Q-STAR probe to the detection of double-stranded DNA and succeeded in obtaining a sequence-specific fluorescence signal [90].

On the other hand, a new releasing system using an azide-based immolative linker was reported by Winssinger and coworkers [93]. The functional molecules are connected to these linkers via carbonate or carbonyl linkage and are released by a reduction reaction in the presence of template. These authors examined the bioactive molecule (estradiol or doxorubicin) as a functional molecule and reported that this system has broad utility.

## 6. Templated Peptide Chemical Reaction

Seitz and coworkers reported a method for single-base mismatch detection that used a native chemical ligation on the template [94,95]. This method consists of two PNA probes: one is the donor probe, which is modified by a thioester group at the C terminal, whereas the other is the accepter probe, which is conjugated with a cysteine (Cys) at the N terminal. When two PNA probes are brought into close proximity at the template, the Cys of the accepter attacks the thioester group of the other probe. The resulting thioester intermediate forms a new peptide bond by irreversible *S*→*N* acyl shift and produces the ligation product. The reactivity of these probes with a matched sequence was 3,000 times faster than it was with a single-base mismatch sequence [95]. However, product inhibition occurred; thus, the turnover number was limited to 51 times in 24 h [96]. Therefore, these authors improved the inhibition of the product by using isocysteine (*i*Cys) instead of Cys [96,97]. As a result, the turnover number significantly improved up to 226 in 24 h [96].

**Figure 7:**
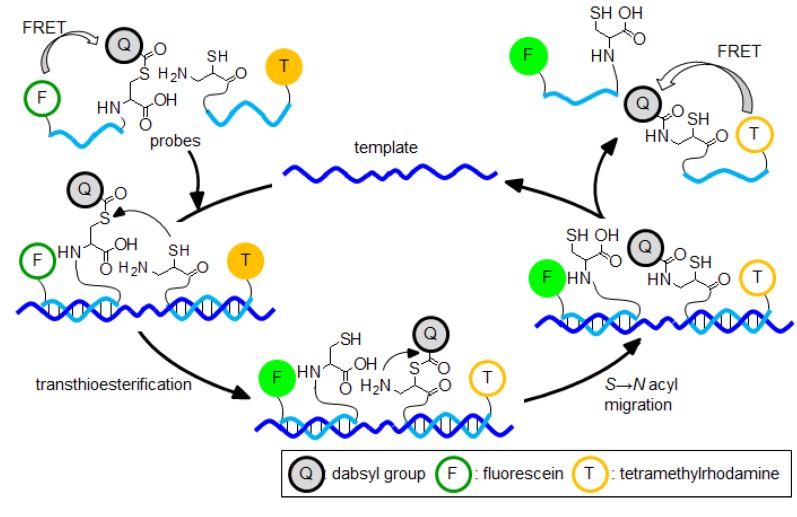
Catalytic cycle of the templated transfer of the reporter group.

Furthermore, Seitz and coworkers reported a signal-amplification system that uses a transfer reaction of the reporter group between the donor probe and the accepter probe in a native-chemical-ligation-like fashion (Figure 7) [98,99]. The donor probe labeled the quencher by the thioester bond at the C terminal. The accepter probe is conjugated with *i*Cys at the N terminal. The transfer reaction proceeds only in the presence of complementary templates. In the case of this reaction, the new linkage did not occur between the donor probe and the accepter probe; thus, the catalytic cycles efficiently rotated. The reaction of these probes with a matched template is completed within 30 min. In contrast, in the presence of a single-base mismatch, the initial rate of the reaction decreased by 44 times. The turnover number of the catalytic reaction was 402 within 24 h, with a maximum observed in the template reaction under the isothermal conditions [98]. Furthermore, these authors developed a double signal amplification method that is performed by combining a DNA catalyst reporter transfer reaction and ELISA [100]. This method enabled the detection of 500 attomol of HIV-I RNA. In addition, Seitz and coworkers reported the synthesis of a bioactive peptide using a DNA template-catalyzed transfer reaction [101,102].

## 7. Other Templated Reactions

**Figure 8:**
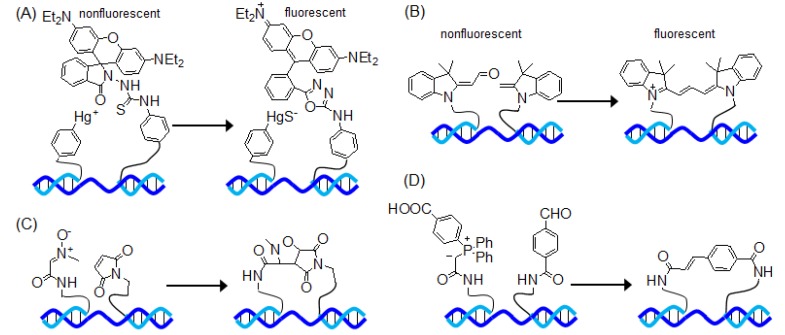
Schematic representation of (**A**) organomercury-promoted oxadiazole forming reaction; (**B**) aldolization-elimination reaction; (**C**) 1,3-dipolar cycloaddition reaction; and (**D**) Wittig olefination reaction.

Franzini and Kool reported a nucleic acid detection system based on an organomercury-activated templated reaction [103]. This system consisted of two probes: one probe is conjugated to *p*-mercuribenzoate groups and the other is conjugated to rhodamine B phenylthiosemicarbazide, which is the analytical reagent used for the detection of Hg^2+^. In the presence of template, the thiosemicarbazide moiety was attacked by the Hg^2+^, was converted to the oxadiazole type, and induced fluorescence signals (Figure 8A). These probes have been used to discriminate single-base mismatches in a few minutes.

Ladame and coworkers reported the detection of DNA hairpin and G-quadruplex structures [104,105]. This system consisted of the aldehyde and indole probes. In the presence of the target high-order DNA structure, the schiff base free-templated aldolization-elimination reaction between the aldehyde probe and the indole probe leads to the formation of a fluorescent trimethine cyanine dye (Figure 8B). These probes have been used to sense a high-order DNA structure and generated fluorescence signals.

Liu and coworkers reported a DNA-templated synthesis method based on 1,3-dipolar cycloaddition or Wittig olefination (Figure 8C,D) [106]. Furthermore, they examined in detail the influence of distance (between the reactive groups) and of the second structure (for example, the end-of-helix or hairpin architectures) on the templated reaction [106,107,108].

**Figure 9:**
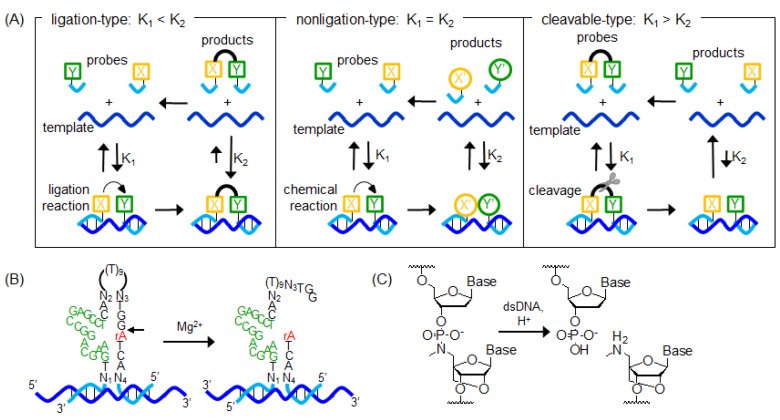
Schematic representation of (**A**) the thermodynamic cycle of templated reaction; (**B**) the self-cleaving DNAzyme; and (**C**) the cleavage of a P3'→N5' phosphoramidate linkage.

The dissociation of the product from the template should be uninhibited to increase the turnover of OTRs. OTRs are of three types: ligation, nonligation, or cleavable types (Figure 9A). For the ligation-type reaction, the binding affinity of the reaction product to the template is increased compared with the binding affinity of the probes, so that the cycle of OTRs is strongly inhibited at the dissociation step. In contrast, for the nonligation-type reaction, the binding affinity of the reaction product is invariable. Therefore, these probes do not occur at the product inhibition. However, the products of the nonligation-type reaction are not promoted at the dissociation. For the cleavable-type reaction, two methods that promoted dissociation under the isothermal condition have been reported: one method included the DNAzyme in the structure of the probe and the other method included the P3'→N5' phosphoramidate linkage in the probe. In these methods, after the probe is bound to the template, the probe is cleaved without the help of any other reagents. As a result, the binding affinity of the product is decreased when compared to the probe, so that the turnover of OTRs is promoted. Sando and coworkers reported a nucleic acid detection method by utilizing the DNAzyme (Figure 9B) [109,110]. When the probe is bound to a template, the DNAzyme motif of the probe is activated, which cleaves the probe at the scission site. These authors succeeded in imaging 16S rRNA in fixed *E. coli* cells using this probe [110]. On the other hand, Obika and coworkers reported a detection method by utilizing the acid-mediate cleavage of P3'→N5' phosphoramidate linkage (Figure 9C) [111,112,113]. They synthesized a probe containing the 5'-amino-2'-O, 4'-*C*-methylene bridged nucleic acid at the center. In the presence of template, the initial rate of cleavage reaction of P3'→N5' phosphoramidate linkage was about 20 times higher than observed in the absence of template. Moreover, they have reported that the detection of DNA duplex was possible by using this probe.

## 8. Conclusions

OTRs stimulate chemists to develop and apply new chemistries for nucleic-acid sensing. One of the advantages of OTRs is the accumulation of product via multiple reactions, leading to signal amplification. Signal amplification of RNAs in living cells is quite challenging because of their low copy number. Previous methods based on S_N_2 or Staudinger reaction have succeeded in detecting ribosomal RNAs or housekeeping genes, which have a comparatively greater level of expression. However, the detection of biologically important mRNAs in living cells remains challenging. Therefore, further improvements of OTR probes are needed to achieve high sensitivity. Recent results have shown that nonligation-type or cleavable-type reactions may represent efficient turnover reactions that can be used to relieve product inhibition. Moreover, results of experiments using nonligation-type probes indicate that fluorogenic molecules that generate fluorescence triggered by a chemical reaction exhibited an S/B ratio that was higher than the FRET mechanism. Based on these investigations, a more advanced probe that is capable of ultrasensitive detection is expected to combine efficient turnover ability and a fluorogenic system with a high S/B ratio.

## References

[1] Silverman A.P., Kool E.T. (2006). Detecting RNA and DNA with templated chemical reactions. Chem. Rev..

[2] Grossmann T.N., Strohbach A., Seitz O. (2008). Achieving turnover in DNA-templated reactions. ChemBioChem.

[3] Lewis R.J., Hanawalt P.C. (1982). Ligation of oligonucleotides by pyrimidine dimers—A missing “link” in the origin of life?. Nature.

[4] Churchill M.E., Peak J.G., Peak M.J. (1991). Correlation between cell survival and DNA single-strand break repair proficiency in the Chinese hamster ovary cell lines AA8 and EM9 irradiated with 365-nm ultraviolet-A radiation. Photochem. Photobiol..

[5] Albagli D., van Atta R., Cheng P., Huan B.F., Wood M.L. (1999). Chemical amplification (CHAMP) by a continuous, self-replicating oligonucleotide-based system. J. Am. Chem. Soc..

[6] Woo J., Hopkins P.B. (1991). Template directed modification of single stranded DNA by psoralen-tethered oligonucleotides: Sites of photoadduct formation analyzed by sequence-specific and sequence-random cleavage. J. Am. Chem. Soc..

[7] Lewis F.D., Wu T.F., Burch E.L., Bassani D.M., Yang J.S., Schneider S., Jager W., Letsinger R.L. (1995). Hybrid oligonucleotides containing stilbene units. Excimer fluorescence and photodimerization. J. Am. Chem. Soc..

[8] Letsinger R.L., Wu T.F. (1994). Control of excimer emission and photochemistry of stilbene units by oligonucleotide hybridization. J. Am. Chem. Soc..

[9] Liu J.Q., Taylor J.S. (1996). Remarkable photoreversal of a thio analog of the dewar valence isomer of the (6-4) photoproduct of DNA to the parent nucleotides. J. Am. Chem. Soc..

[10] Zhao X.D., Liu J.Q., Hsu D.S., Zhao S.Y., Taylor J.S., Sancar A. (1997). Reaction mechanism of (6-4) photolyase. J. Biol. Chem..

[11] Liu J.Q., Taylor J.S. (1998). Template-directed photoligation of oligodeoxyribonucleotides via 4-thiothymidine. Nucleic Acids Res..

[12] Fujimoto K., Matsuda S., Takahashi N., Saito I. (2000). Template-directed photoreversible ligation of deoxyoligonucleotides via 5-vinyldeoxyuridine. J. Am. Chem. Soc..

[13] Fujimoto K., Matsuda S., Ogawa N., Hayashi M., Saito I. (2000). Template-directed reversible photocircularization of DNA via 5-vinyldeoxycytidine. Tetrahedron Lett..

[14] Fujimoto K., Matsuda S., Hayashi M., Saito I. (2000). Reversible DNA photocircularization on triple helix: Effect of vinyl substituent on base stacking. Tetrahedron Lett..

[15] Fujimoto K., Ogawa N., Hayashi M., Matsuda S., Saito I. (2000). Template directed photochemical synthesis of branched oligodeoxynucleotides via 5-carboxyvinyldeoxyuridine. Tetrahedron Lett..

[16] Fujimoto K., Matsuda S., Yoshimura Y., Matsumura T., Hayashi M., Saito I. (2006). Site-specific transition of cytosine to uracil via reversible DNA photoligation. Chem. Commun..

[17] Fujimoto K., Matsuda S., Yoshimura Y., Ami T., Saito I. (2007). Reversible photopadlocking on double-stranded DNA. Chem. Commun..

[18] Ogasawara S., Fujimoto K. (2005). Solution of a SAT problem on a photochemical DNA computer. Chem. Lett..

[19] Yoshimura Y., Noguchi Y., Sato H., Fujimoto K. (2006). Template-directed DNA photoligation in rapid and selective detection of RNA point mutations. ChemBioChem.

[20] Fujimoto K., Yoshino H., Ami T., Yoshimura Y., Saito I. (2008). A light-controlled reversible DNA photoligation via carbazole-tethered 5-carboxyvinyluracil. Org. Lett..

[21] Nakamura S., Ogasawara S., Matuda S., Saito I., Fujimoto K. (2011). Template directed reversible photochemical ligation of oligodeoxynucleotides. Molecules.

[22] Saito I., Miyauchi Y., Saito Y., Fujimoto K. (2005). Template-directed photoreversible ligation of DNA via 7-carboxyvinyl-7-deaza-2'-deoxyadenosine. Tetrahedron Lett..

[23] Yoshimura Y., Okamura D., Ogino M., Fujimoto K. (2006). Highly selective and sensitive template-directed photoligation of DNA via 5-carbamoylvinyl-2'-deoxycytidine. Org. Lett..

[24] Ami T., Fujimoto K. (2008). Click chemistry as an efficient method for preparing a sensitive DNA probe for photochemical ligation. ChemBioChem.

[25] Ami T., Yoshimura Y., Matsuzaki T., Fujimoto K. (2009). Photochemical ligation of DNA probe prepared in click chemistry. J. Photopolym. Sci. Technol..

[26] Ogino M., Yoshimura Y., Nakazawa A., Saito I., Fujimoto K. (2005). Template-directed DNA photoligation via alpha-5-cyanovinyldeoxyuridine. Org. Lett..

[27] Ami T., Fujimoto K. (2006). Fluorescence labeling of DNA based on photochemical ligation. Sci. Technol. Adv. Mater..

[28] Fujimoto K., Konishi-Hiratsuka K., Sakamoto T., Yoshimura Y. (2010). Site-specific photochemical RNA editing. Chem. Commun..

[29] Fujimoto K., Konishi-Hiratsuka K., Sakamoto T., Yoshimura Y. (2010). Site-specific cytosine to uracil transition by using reversible DNA photo-crosslinking. ChemBioChem.

[30] Ihara T., Fujii T., Mukae M., Kitamura Y., Jyo A. (2004). Photochemical ligation of DNA conjugates through anthracene cyclodimer formation and its fidelity to the template sequences. J. Am. Chem. Soc..

[31] Arslan P., Ihara T., Mukae M., Jyo A. (2008). The effect of local structural disruption on the yield of photochemical ligation between anthracene-oligonucleotide conjugates. Anal. Sci..

[32] Mukae M., Ihara T., Tabara M., Jyo A. (2009). Anthracene-DNA conjugates as building blocks of designed DNA structures constructed by photochemical reactions. Org. Biomol. Chem..

[33] Arslan P., Jyo A., Ihara T. (2010). Reversible circularization of an anthracene-modified DNA conjugate through bimolecular triplex formation and its analytical application. Org. Biomol. Chem..

[34] Becker H.D. (1993). Unimolecular photochemistry of anthracenes. Chem. Rev..

[35] Bouas-Laurent H., Castellan A., Desvergne J.P., Lapouyade R. (2000). Photodimerization of anthracenes in fluid solution: Structural aspects. Chem. Soc. Rev..

[36] Pasternak K., Pasternak A., Gupta P., Veedu R.N., Wengel J. (2011). Photoligation of self-assembled DNA constructs containing anthracene-functionalized 2'-amino-LNA monomers. Bioorg. Med. Chem..

[37] Lee H.M., Larson D.R., Lawrence D.S. (2009). Illuminating the chemistry of life: Design, synthesis, and applications of “caged” and related photoresponsive compounds. ACS Chem. Biol..

[38] Okamoto A., Tanabe K., Inasaki T., Saito I. (2003). Phototriggered drug release from functionalized oligonucleotides by a molecular beacon strategy. Angew. Chem. Int. Edit..

[39] Tanabe K., Nakata H., Mukai S., Nishimoto S. (2005). Modulated drug release from the stem-and-loop structured oligodeoxynucleotide upon UV-A irradiation in the presence of target DNA. Org. Biomol. Chem..

[40] Rothlingshofer M., Gorska K., Winssinger N. (2011). Nucleic acid-templated energy transfer leading to a photorelease reaction and its application to a system displaying a nonlinear response. J. Am. Chem. Soc..

[41] Clo E., Snyder J.W., Voigt N.V., Ogilby P.R., Gothelf K.V. (2006). DNA-programmed control of photosensitized singlet oxygen production. J. Am. Chem. Soc..

[42] Clo E., Snyder J.W., Ogilby P.R., Gothelf K.V. (2007). Control and selectivity of photosensitized singlet oxygen production: Challenges in complex biological systems. ChemBioChem.

[43] Jacobsen M.F., Clo E., Mokhir A., Gothelf K.V. (2007). Model systems for activation of nucleic acid encoded prodrugs. ChemMedChem.

[44] Arian D., Clo E., Gothelf K.V., Mokhir A. (2010). A nucleic acid dependent chemical photocatalysis in live human cells. Chem. Eur. J..

[45] Ma Z.C., Taylor J.S. (2000). Nucleic acid-triggered catalytic drug release. Proc. Natl. Acad. Sci. USA.

[46] Ma Z.C., Taylor J.S. (2001). Nucleic acid triggered catalytic drug and probe release: A new concept for the design of chemotherapeutic and diagnostic agents. Bioorg. Med. Chem..

[47] Ma Z.C., Taylor J.S. (2003). PNA-based RNA-triggered drug-releasing system. Bioconjug. Chem..

[48] Cai J.F., Li X.X., Taylor J.S. (2005). Improved nucleic acid triggered probe activation through the use of a 5-thiomethyluracil peptide nucleic acid building block. Org. Lett..

[49] Brunner J., Mokhir A., Kraemer R. (2003). DNA-templated metal catalysis. J. Am. Chem. Soc..

[50] Zelder F.H., Brunner J., Kramer R. (2004). DNA-templated catalysis using a metal-cleavable linker. Chem. Commun..

[51] Boll I., Kramer R., Brunner J., Mokhir A. (2005). Templated metal catalysis for single nucleotide specific DNA sequence detection. J. Am. Chem. Soc..

[52] Gryaznov S.M., Letsinger R.L. (1993). Chemical ligation of oligonucleotides in the presence and absence of a template. J. Am. Chem. Soc..

[53] Gryaznov S.M., Schultz R., Chaturvedi S.K., Letsinger R.L. (1994). Enhancement of selectivity in recognition of nucleic acids via chemical autoligation. Nucleic Acids Res..

[54] Herrlein M.K., Letsinger R.L. (1994). Selective chemical autoligation on a double-stranded DNA template. Nucleic Acids Res..

[55] Herrlein M.K., Nelson J.S., Letsinger R.L. (1995). A covalent lock for self-assembled oligonucleotide conjugates. J. Am. Chem. Soc..

[56] Xu Y.Z., Kool E.T. (1999). High sequence fidelity in a non-enzymatic DNA autoligation reaction. Nucleic Acids Res..

[57] Xu Y.Z., Kool E.T. (2000). Rapid and selective selenium-mediated autoligation of DNA strands. J. Am. Chem. Soc..

[58] Xu Y.Z., Karalkar N.B., Kool E.T. (2001). Nonenzymatic autoligation in direct three-color detection of RNA and DNA point mutations. Nat. Biotechnol..

[59] Gartner Z.J., Liu D.R. (2001). The generality of DNA-templated synthesis as a basis for evolving non-natural small molecules. J. Am. Chem. Soc..

[60] Abe H., Kondo Y., Jinmei H., Abe N., Furukawa K., Uchiyama A., Tsuneda S., Aikawa K., Matsumoto I., Ito Y. (2008). Rapid DNA chemical ligation for amplification of RNA and DNA signal. Bioconjug. Chem..

[61] Sando S., Kool E.T. (2002). Quencher as leaving group: Efficient detection of DNA-joining reactions. J. Am. Chem. Soc..

[62] Sando S., Kool E.T. (2002). Imaging of RNA in bacteria with self-ligating quenched probes. J. Am. Chem. Soc..

[63] Sando S., Abe H., Kool E.T. (2004). Quenched auto-ligating DNAs: Multicolor identification of nucleic acids at single nucleotide resolution. J. Am. Chem. Soc..

[64] Silverman A.P., Kool E.T. (2005). Quenched probes for highly specific detection of cellular RNAs. Trends Biotechnol..

[65] Abe H., Kool E.T. (2004). Destabilizing universal linkers for signal amplification in self-ligating probes for RNA. J. Am. Chem. Soc..

[66] Abe H., Kool E.T. (2006). Flow cytometric detection of specific RNAs in native human cells with quenched autoligating FRET probes. Proc. Natl. Acad. Sci. USA.

[67] Silverman A.P., Baron E.J., Kool E.T. (2006). RNA-templated chemistry in cells: Discrimination of *Escherichia*, *Shigella* and *Salmonella* bacterial strains with a new two-color FRET strategy. ChemBioChem.

[68] Silverman A.P., Kool E.T. (2005). Quenched autoligation probes allow discrimination of live bacterial species by single nucleotide differences in rRNA. Nucleic Acids Res..

[69] Miller G.P., Silverman A.P., Kool E.T. (2008). New, stronger nucleophiles for nucleic acid-templated chemistry: Synthesis and application in fluorescence detection of cellular RNA. Bioorg. Med. Chem..

[70] Shibata A., Abe H., Ito M., Kondo Y., Shimizu S., Aikawa K., Ito Y. (2009). DNA templated nucleophilic aromatic substitution reactions for fluorogenic sensing of oligonucleotides. Chem. Commun..

[71] Lemieux G.A., de Graffenried C.L., Bertozzi C.R. (2003). A fluorogenic dye activated by the Staudinger ligation. J. Am. Chem. Soc..

[72] Prescher J.A., Bertozzi C.R. (2005). Chemistry in living systems. Nat. Chem. Biol..

[73] El-Sagheer A.H., Brown T. (2010). Click chemistry with DNA. Chem. Soc. Rev..

[74] Le Droumaguet C., Wang C., Wang Q. (2010). Fluorogenic click reaction. Chem. Soc. Rev..

[75] Jewett J.C., Bertozzi C.R. (2010). Cu-free click cycloaddition reactions in chemical biology. Chem. Soc. Rev..

[76] Cai J.F., Li X.X., Yue X., Taylor J.S. (2004). Nucleic acid-triggered fluorescent probe activation by the staudinger reaction. J. Am. Chem. Soc..

[77] Pianowski Z.L., Winssinger N. (2007). Fluorescence-based detection of single nucleotide permutation in DNA via catalytically templated reaction. Chem. Commun..

[78] Pianowski Z., Gorska K., Oswald L., Merten C.A., Winssinger N. (2009). Imaging of mRNA in live cells using nucleic acid-templated reduction of azidorhodamine probes. J. Am. Chem. Soc..

[79] Gorska K., Keklikoglou I., Tschulena U., Winssinger N. (2011). Rapid fluorescence imaging of miRNAs in human cells using templated Staudinger reaction. Chem. Sci..

[80] Abe H., Wang J., Furukawa K., Oki K., Uda M., Tsuneda S., Ito Y. (2008). A reduction-triggered fluorescence probe for sensing nucleic acids. Bioconjug. Chem..

[81] Furukawa K., Abe H., Wang J., Uda M., Koshino H., Tsuneda S., Ito Y. (2009). Reduction-triggered red fluorescent probes for dual-color detection of oligonucleotide sequences. Org. Biomol. Chem..

[82] Franzini R.M., Kool E.T. (2008). 7-Azidomethoxy-coumarins as profluorophores for templated nucleic acid detection. ChemBioChem.

[83] Furukawa K., Abe H., Hibino K., Sako Y., Tsuneda S., Ito Y. (2009). Reduction-triggered fluorescent amplification probe for the detection of endogenous rnas in living human cells. Bioconjug. Chem..

[84] Shibata A., Abe H., Furukawa K., Tsuneda S., Ito Y. (2009). Reduction-triggered fluorescence probe for peptide-templated reactions. Chem. Pharm. Bull..

[85] Furukawa K., Abe H., Tamura Y., Yoshimoto R., Yoshida M., Tsuneda S., Ito Y. (2011). Fluorescence detection of intron lariat RNA with reduction-triggered fluorescent probes. Angew. Chem. Int. Edit.

[86] Marras S.A.E., Kramer F.R., Tyagi S. (2002). Efficiencies of fluorescence resonance energy transfer and contact-mediated quenching in oligonucleotide probes. Nucleic Acids Res..

[87] Rothlingshofer M., Gorska K., Winssinger N. (2012). Nucleic acid templated uncaging of fluorophores using ru-catalyzed photoreduction with visible light. Org. Lett..

[88] Chen Y.Y., Kamlet A.S., Steinman J.B., Liu D.R. (2011). A biomolecule-compatible visible-light-induced azide reduction from a DNA-encoded reaction-discovery system. Nat. Chem..

[89] Franzini R.M., Kool E.T. (2009). Efficient nucleic acid detection by templated reductive quencher release. J. Am. Chem. Soc..

[90] Li H., Franzini R.M., Bruner C., Kool E.T. (2010). Templated chemistry for sequence-specific fluorogenic detection of duplex DNA. ChemBioChem.

[91] Franzini R.M., Kool E.T. (2011). Two successive reactions on a DNA template: A strategy for improving background fluorescence and specificity in nucleic acid detection. Chem. Eur. J..

[92] Franzini R.M., Kool E.T. (2011). Improved templated fluorogenic probes enhance the analysis of closely related pathogenic bacteria by microscopy and flow cytometry. Bioconjugate Chem..

[93] Gorska K., Manicardi A., Barluenga S., Winssinger N. (2011). DNA-templated release of functional molecules with an azide-reduction-triggered immolative linker. Chem. Commun..

[94] Ficht S., Mattes A., Seitz O. (2004). Single-nucleotide-specific PNA-peptide ligation on synthetic and PCR DNA templates. J. Am. Chem. Soc..

[95] Ficht S., Dose C., Seitz O. (2005). As fast and selective as enzymatic ligations: Unpaired nucleobases increase the selectivity of DNA-controlled native chemical PNA ligation. ChemBioChem.

[96] Dose C., Ficht S., Seitz O. (2006). Reducing product inhibition in DNA-template-controlled ligation reactions. Angew. Chem. Int. Ed..

[97] Dose C., Seitz O. (2008). Single nucleotide specific detection of DNA by native chemical ligation of fluorescence labeled PNA-probes. Bioorg. Med. Chem..

[98] Grossmann T.N., Seitz O. (2006). DNA-catalyzed transfer of a reporter group. J. Am. Chem. Soc..

[99] Grossmann T.N., Seitz O. (2009). Nucleic acid templated reactions: Consequences of probe reactivity and readout strategy for amplified signaling and sequence selectivity. Chem. Eur. J..

[100] Grossmann T.N., Roglin L., Seitz O. (2008). Target-catalyzed transfer reactions for the amplified detection of RNA. Angew. Chem. Int. Edit..

[101] Erben A., Grossmann T.N., Seitz O. (2011). DNA-triggered synthesis and bioactivity of proapoptotic peptides. Angew. Chem. Int. Edit..

[102] Erben A., Grossmann T.N., Seitz O. (2011). DNA-instructed acyl transfer reactions for the synthesis of bioactive peptides. Bioorg. Med. Chem. Lett..

[103] Franzini R.M., Kool E.T. (2008). Organometallic activation of a fluorogen for templated nucleic acid detection. Org. Lett..

[104] Meguellati K., Koripelly G., Ladame S. (2010). DNA-templated synthesis of trimethine cyanine dyes: A versatile fluorogenic reaction for sensing G-quadruplex formation. Angew. Chem. Int. Edit..

[105] Koripelly G., Meguellati K., Ladame S. (2010). Dual sensing of hairpin and quadruplex DNA structures using multicolored peptide nucleic acid fluorescent probes. Bioconjug. Chem..

[106] Gartner Z.J., Grubina R., Calderone C.T., Liu D.R. (2003). Two enabling architectures for DNA-templated organic synthesis. Angew. Chem. Int. Edit..

[107] Calderone C.T., Liu D.R. (2004). Nucleic-acid-templated synthesis as a model system for ancient translation. Curr. Opin. Chem. Biol..

[108] Li X.Y., Liu D.R. (2004). DNA-Templated organic synthesis: Nature’s strategy for controlling chemical reactivity applied to synthetic molecules. Angew. Chem. Int. Edit..

[109] Sando S., Sasaki T., Kanatani K., Aoyama Y. (2003). Amplified nucleic acid sensing using programmed self-cleaving DNAzyme. J. Am. Chem. Soc..

[110] Sando S., Narita A., Sasaki T., Aoyama Y. (2005). . Locked TASC probes for homogeneous sensing of nucleic acids and imaging of fixed *E-coli* cells. Org. Biomol. Chem..

[111] Obika S., Tomizu M., Negoro Y., Orita A., Nakagawa O., Imanishi T. (2007). Double-stranded DNA-templated oligonucleotide digestion triggered by triplex formation. ChemBioChem.

[112] Ito K.R., Kodama T., Tomizu M., Negoro Y., Orita A., Osaki T., Hosoki N., Tanaka T., Imanishi T., Obika S. (2010). Double-stranded DNA-templated cleavage of oligonucleotides containing a P3'→N5' linkage triggered by triplex formation: The effects of chemical modifications and remarkable enhancement in reactivity. Nucleic Acids Res..

[113] Ito K.R., Kodama T., Makimura F., Hosoki N., Osaki T., Orita A., Imanishi T., Obika S. (2011). Cleavage of oligonucleotides containing a P3'→N5' phosphoramidate linkage mediated by single-stranded oligonucleotide templates. Molecules.

